# Tailoring Robust Quantum Anomalous Hall Effect via Entropy‐Engineering

**DOI:** 10.1002/adma.202503319

**Published:** 2025-06-03

**Authors:** Syeda Amina Shabbir, Frank Fei Yun, Muhammad Nadeem, Xiaolin Wang

**Affiliations:** ^1^ Institute for Superconducting and Electronic Materials (ISEM), Faculty of Engineering and Information Sciences (EIS) University of Wollongong Wollongong New South Wales 2525 Australia

**Keywords:** dirac half‐metals, high entropy materials, quantum anomalous hall effect, quantum materials, spin gapless semiconductors, topological transport

## Abstract

The development of quantum materials and the tailoring of their functional properties is of fundamental interest in materials science. Here, a new design concept is proposed for the robust quantum anomalous Hall (QAH) effect via entropy engineering in 2D magnets. As a prototypical example, the configurational entropy of monolayer transition metal trihalide VCl_3_ is manipulated by incorporating four different transition‐metal cations [Ti,Cr,Fe,Co] into the honeycomb structure made of vanadium, such that all in‐plane mirror symmetries, inversion and/or roto‐inversion are broken. Monolayer VCl_3_ is a ferromagnetic Dirac half‐metal in which spin‐polarized Dirac dispersion at valley momenta is accompanied by bulk states at the Γ‐point and thus the spin‐orbit interaction‐driven QAH phase does not exhibit fully gapped bulk band dispersion. Entropy‐driven bandstructure renormalization, especially band flattening in combination with red‐ and blue‐shifts at different momenta of the Brillouin zone and crystal‐field effects, transforms Dirac half‐metal to a Dirac spin‐gapless semiconductor and leads to a robust QAH phase with fully gapped bulk band dispersion and, thus, a purely topological edge state transport without mixing with dissipative bulk channels. These findings provide a paradigm for designing entropy‐engineered 2D materials for the realization of robust QAH effect and quantum device applications.

## Graphical abstract

Bandstructure renormalization, without (w/o) spin‐orbit interaction (SOI) (left) and with SOI (right), in the low‐energy Dirac bands of zero‐entropy (purple) and entropic (green) VCl3 monolayer (ML) with long‐range ordering (LRO).

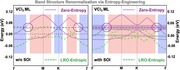

## 1 Introduction

Spin‐gapless semiconductors (SGSs)^[^ ^1^, ^2^ ^]^ are characterized by gapless dispersion in one of the spin sectors while a gapped spectrum in the other spin sector. With this unique characteristic, SGSs bridge the gap between magnetic semiconductors^[^ ^3^ ^]^ and magnetic half‐metals.^[^ ^4^, ^5^ ^]^ SGSs also serve as a fundamental ingredient for theoretical understanding and experimental realization of various exotic quantum phases such as the quantum anomalous Hall (QAH) effect,^[^ ^2^, ^6^, ^7^ ^]^ new (quantum) anomalous spin Hall effects,^[^ ^8^ ^]^ and topological nodal line spin‐gapless semimetals.^[^ ^2^, ^9^ ^]^ In addition, because of their intriguing electronic, magnetic and optical features, SGSs are promising candidates for quantum device applications, both with conventional bulk transport in tunneling junctions featuring large spin filtering and high tunneling magnetoresistance and with dissipationless topological edge state transport.^[^ ^2^, ^10^, ^11^, ^12^, ^13^, ^14^ ^]^

After the seminal proposal for SGSs in 2008,^[^ ^1^ ^]^ SGSs have been predicted in a variety of materials classes.^[^ ^2^ ^]^ However, the experimental realization of SGSs and spin‐orbit interaction (SOI) driven QAH effect has been hindered on several fronts. For instance, while indirect SGSs with parabolic dispersion have been confirmed, experimental confirmation of direct SGSs with Dirac/parabolic dispersion is limited. Similarly, other than intrinsic SGSs, various QAH materials have been fabricated and characterized, but with low Curie temperature, small topological bandgap, and the presence of dissipative channels along the dissipationless chiral edge states. These challenges can be evaded with experimentally accessible direct SGSs where intrinsic magnetism and SOI could open a topologically nontrivial bandgap featuring a robust QAH effect. The search for direct SGSs and the corresponding high‐temperature (Curie) robust QAH effect motivates bandstructure engineering in half‐metallic magnets and magnetic semiconductors.

**Figure 1:**
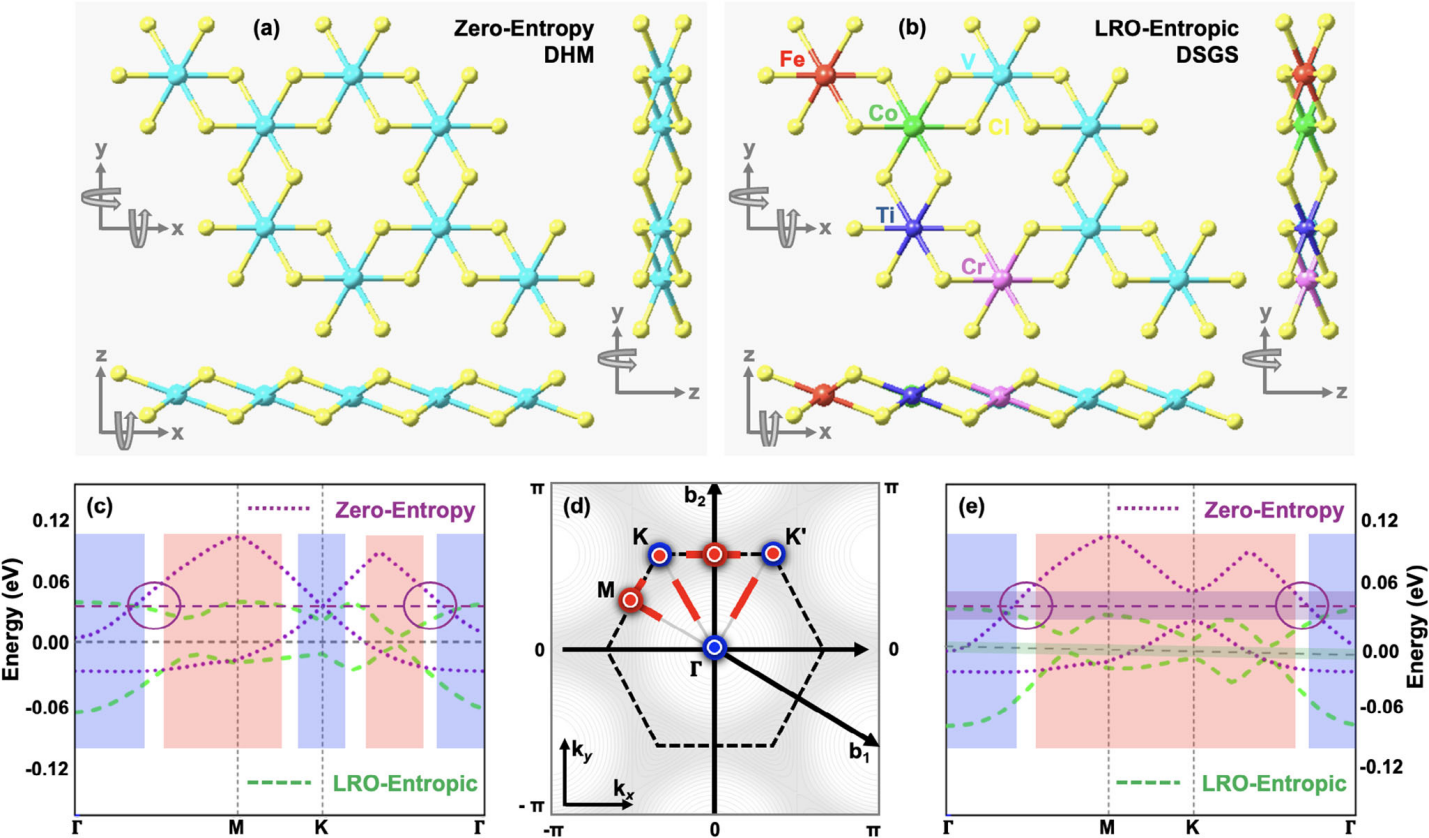
Design concept and bandstructure renormalization via entropy engineering. a,b) Lattice structure of zero‐entropy V_8_Cl_24_ monolayer (a) and entropic V_4_TiCrFeCoCl_24_ monolayer (b) with top and side views. c–e) Low‐energy Dirac bands of zero‐entropy V_8_Cl_24_ monolayer (purple) and entropic V_4_TiCrFeCoCl_24_ monolayer (green) without SOI (c) and with SOI (e). Here, red and blue regions, respectively, represent entropy‐driven red and blue shifts in the energy spectrum. The first Brillouin zone showing red and blue shifts at/along high‐symmetry points/lines where large (small) spheres indicate red/blue shift in the absence (presence) of SOI (d). Although the energy shift remains red and blue across the M and Γ points, respectively, the energy dispersion across valleys K/K′ exhibits a blue shift in the absence of SOI while a red shift in the presence of SOI. These entropy‐driven red and blue shifts in the energy spectrum lead to the bandstructure renormalization.

Here we propose a new design concept for SGSs through entropy engineering. Entropy engineering is a new concept of materials design rendering the entropy‐dominated phase stabilization and tailoring of functional properties.^[^ ^15^, ^16^, ^17^, ^18^ ^]^ Due to the direct bandgap closing and an inherent connection of Dirac half‐metals (DHMs) with Dirac SGSs (DSGSs), we consider monolayer vanadium trichloride VCl_3_ as a material platform and manipulated its configurational entropy by incorporating four different transition metal (TM) atoms [M′: Ti, Cr, Fe, Co] in the honeycomb structure formed by vanadium cations, as shown in **Figure** **1**a,b. The M′ cations are substituted by vanadium atoms such that all in‐plane mirror symmetries, inversion, and/or roto‐inversion, are broken. Entropy‐dominated effects on the electronic and spintronic properties of entropic V_0.5_(TiCrFeCo)_0.5_Cl_3_ monolayer are studied using first‐principles calculations. The entropy manipulation results in several intriguing features, such as band flattening due to red and blue shifts at different momenta of the Brillouin zone and a momentum shift of Dirac points due to the modification of in‐plane crystal fields, as shown in Figure 1c–e. As a consequence of band flattening and crystal‐field effects, VCl_3_ monolayer undergoes a major bandstructure renormalization. First, renormalization of the entropy‐driven band structure transforms the DHM phase in the VCl_3_ monolayer to a DSGS phase in the entropic V_0.5_(TiCrFeCo)_0.5_Cl_3_ monolayer, referred here as entropy‐engineered DSGS (EE‐DSGS). Second, when SOI is activated, a nontrivial bandgap leads to a robust QAH phase in V_0.5_(TiCrFeCo)_0.5_Cl_3_ monolayer. The robustness of the QAH phase is derived from the bandstructure renormalization; a maximal blue shift at the Γ‐point disentangles chiral edge states lying in the QAH gap from the bulk states and thus assures dissipationless topological transport via chiral edge states.

## 2 Methods and Results

All the computations are performed using the Vienna ab initio simulation package (VASP)^[^ ^19^, ^20^ ^]^ within the generalized gradient approximation (GGA), employing the Perdew–Burke–Ernzerhof (PBE) exchange correlation functional.^[^ ^21^ ^]^ Electronic and nucleic interactions are explained using the projector augmented wave (PAW)^[^ ^22^ ^]^ technique. The energy criterion is set to 10^−5^ ev, while the atom force convergence is equal to 0.01 eV Å^−1^. Moreover, a PW (plane wave) kinetic energy cutoff is set to 500 eV. A 2 × 2 × 1 supercell is built with vacuum spacing equal to 15 Åalong the normal direction so that inter‐layer interaction is prevented. The Brillouin zone was sampled using a 2 × 2 × 1 and 11 × 11 × 1 Gamma‐centered Monkhorst–Pack grids^[^ ^23^ ^]^ for optimization and electronic structure calculations. The unfolding of bands in VCl_3_ 2 × 2 supercell is performed using the VASP band unfolding package, VaspBandUnfolding.^[^ ^24^ ^]^ In addition, orbital‐resolved band structures are calculated using vaspkit software^[^ ^25^ ^]^ and wanniertools software package is used to calculate edge states.^[^ ^26^ ^]^

Configurational entropy (*S*_conf_) is a controllable parameter, and the ‘level of entropy’ within the high entropy matrix could be tuned to achieve desired system functionalities. That is, an interplay between a global disorder and a local disorder allows to explore materials' space exhibiting on‐demand functional properties. It is important to note that neither a high configurational entropy measuring a degree of disorder nor a compositional complexity relying on diverse cations/anions could necessarily enhance functionalities, but a local environment determines material's electronic and magnetic properties via local interactions. In a high entropy configuration, the cation sites are randomly occupied by five or more TM atoms with equiatomic ratios such that *S*_conf_ ⩾ 1.5*R*, where *R* is the gas constant. The presence of clustering, phase separation, short‐range ordering (SRO), or long‐range ordering (LRO), lowers the entropy level from its maximal value, which is due to complete randomness, to its intermediate (1*R* ⩾ *S*_conf_ < 1.5*R*) or low (0 < *S*_conf_ < 1*R*) values.

The ‘level of entropy’ and the type of ordering may depend upon the interplay between kinetic (favorable activation energy) and thermodynamic (favorable Gibbs free energy) control of the phase transition from the synthesis temperature to the thermodynamic equilibrium at room temperature.^[^ ^27^ ^]^ A high entropy system typically synthesized at a high temperature could be stabilized to yield the desired final state the system exhibits by optimizing the thermal history; synthesis temperature, cooling rate, and equilibration time. That is, high entropy of a multi‐component system with random atomic occupancy could be retained through quenching. However, depending on the interatomic interactions, the intermediate equilibration time allows the formation of clustering or SRO, while a longer equilibration time could potentially cause LRO or complete phase separation. In either case, the emergence of the quantum phases inherently dependent on the nearest‐neighbor hopping or local interaction could potentially be realized through bandstructure renormalization via entropy engineering in a multi‐component system. As a seminal case study on the role of entropy engineering in topological quantum matter, here we present a proof‐of‐concept to demonstrate bandstructure renormalization and the emergence of novel quantum phases in multi‐component 2D materials with long‐range ordering.

**Figure 2:**
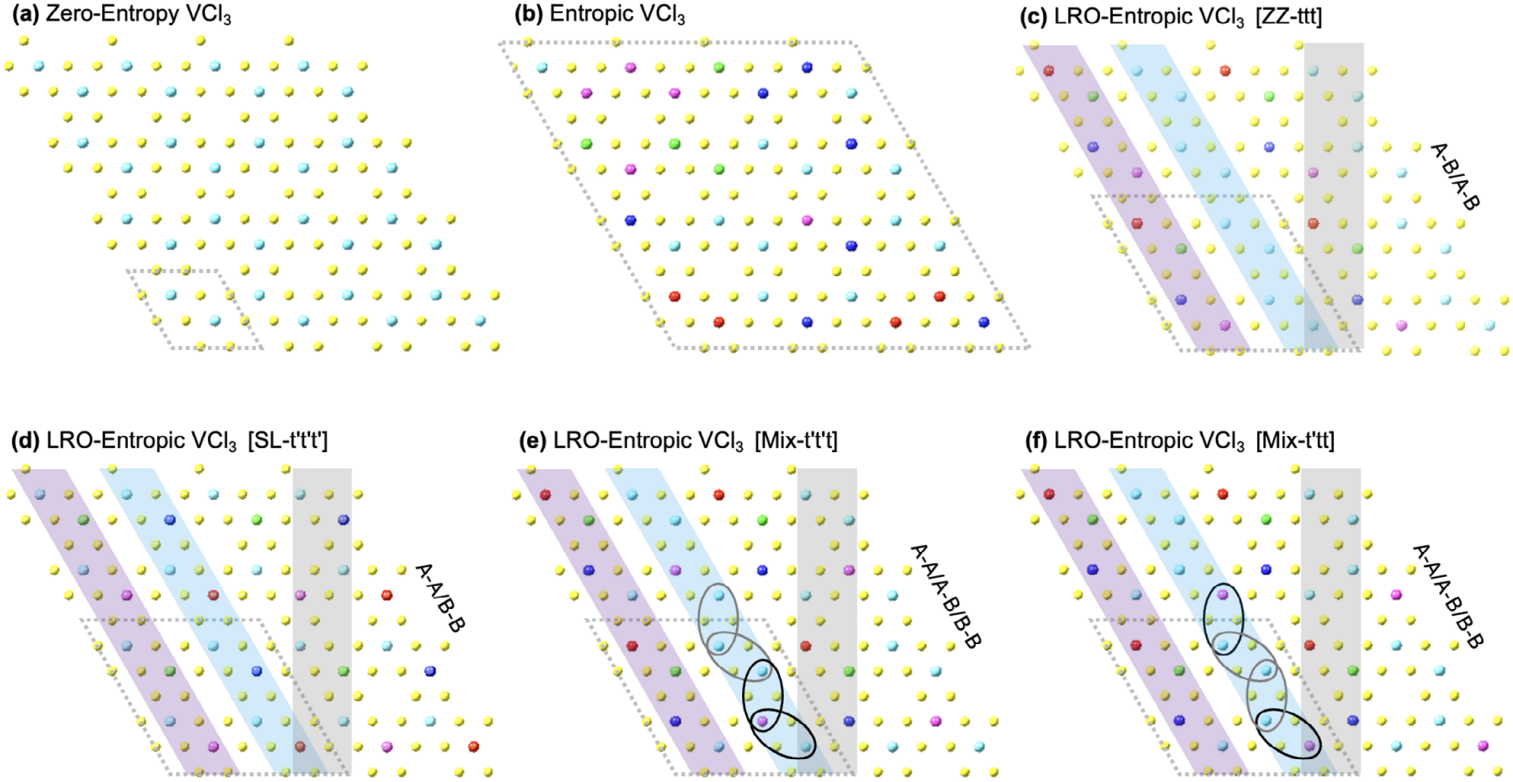
Entropic VCl_3_ monolayer with a long‐range order. a,b) A 4 × 4 supercell of zero‐entropy VCl_3_ (a) and maximal entropic VCl_3_ with a random distribution of TM atoms at A and B sublattice sites of honeycomb structure, without a long‐range order (b). c,d). A 4 × 4 supercell of LRO‐entropic VCl_3_ with a long‐range order along zigzag chains [ZZ‐ttt] with A‐B/A‐B bonds favored by V/M′ atoms (c) and a long‐range order on sublattice sites [SL‐t′t′t′] with A‐A/B‐B bonds favored by V/M′ atoms (d). e,f) A 4 × 4 supercell of LRO‐entropic VCl_3_ with a mixed preference of A‐A/A‐B/B‐B bonds by V/M′ atoms, [Mix‐t′t′t] (e) and [Mix‐t′tt] (f). Panel (f) is same as panel (e), with an exchange of V and Cr sublattice sites, enclosed by black ovals. Here, t and t′, respectively, represent nearest‐neighbor hopping amplitude along V‐V/M′‐M′ bonds and the V‐M′ bonds. The t‐triplet represents the pattern of nearest‐neighbor hopping along zigzag chains, represented by blue and purple strips. Here, vertical gray strips represent the armchair chains.

We consider the VCl_3_ monolayer as a prototypical 2D half‐metallic ferromagnet and its configurational entropy is increased by substituting four vanadium atoms with TM atoms [M′: Ti, Cr, Fe, Co], as shown in **Figure** **2**. The highest level of entropy is traditionally derived from a random distribution of TM atoms at the A and B sublattice sites of the honeycomb structure, Figure 2b. However, the entropy level of V_0.5_(TiCrFeCo)_0.5_Cl_3_ monolayer lowers from its maximal value when the system stabilizes with the long‐range ordering. In this article, as shown in Figure 2c–f, four distinct long‐ranged ordered entropic configurations of V_0.5_(TiCrFeCo)_0.5_Cl_3_ monolayer are studied, displaying an ordering of V/M atoms along (i) zigzag chains with A‐B/A‐B bonds favored by V/M′ atoms, (ii) sublattice sites with A‐A/B‐B bonds favored by V/M′ atoms, and (iii‐iv) a long‐range ordered entropic VCl_3_ with a mixed preference of A‐A/A‐B/B‐B bonds by V/M′ atoms. These LRO‐entropic configurations are labeled as ZZ‐ttt, SL‐t′t′t′, Mix‐t′t′t, and Mix‐t′tt where t and t′ represent the amplitude of nearest‐neighbor hopping along V‐V/M′‐M′ bonds and V‐M′ bonds, respectively. The t‐triplet represents a pattern of nearest‐neighbor hopping between sublattice sites along zigzag chains.

### 2.1 VCl_3_ Monolayer

The monolayer VCl_3_ possess a buckled trilayered structure Cl‐V‐Cl with V atoms sandwiched between Cl atoms, where V atoms form a hexagonal honeycomb structure in which V^3 +^ cations are covalently bonded to six nearest neighboring Cl^−^ anions. Each of the Cl^−^ anions is bonded to two V^3 +^ cations via edge sharing octahedral coordination. Full geometry optimization is carried out on the unit cell of the unchanged VCl_3_ monolayer to relax the structure. The optimized lattice constant obtained is 6.28 Å, which is in good agreement with those previously reported for VCl_3_ monolayer^[^ ^14^, ^28^, ^29^, ^30^ ^]^ and VCl_3_ crystal.^[^ ^31^ ^]^

Several theoretical studies, along with recent experimental observations,^[^ ^32^, ^33^ ^]^ have been reported on VCl_3_. However, mainly due to potentially different and competing ground states associated with different underlying crystalline symmetries and external stimuli,^[^ ^34^, ^35^ ^]^ the VCl_3_ monolayer could display a different electronic structure and magnetic ordering. First‐principles calculations show that the VCl_3_ monolayer could be a ferromagnetic half‐metal,^[^ ^14^, ^28^, ^29^, ^30^ ^]^ a ferromagnetic insulator,^[^ ^36^ ^]^ and a Mott–Hubbard insulator^[^ ^32^ ^]^ with a ferromagnetic Mott insulating ground state followed by an antiferromagnetic transition at *T*_*N*_ = 21.8 K. The seminal experimental study^[^ ^32^ ^]^ that reported the synthesis and characterization of single‐crystalline VCl_3_, accompanied by experimental observations and first‐principles calculations to understand the electronic nature, suggested an intrinsic Mott–Hubbard insulating phase and an extrinsic 2D magnetic polaronic phase. It shows that even a small perturbation could transform an extremely correlated Mott insulating phase into a 2D polaronic phase in the VCl_3_ ionic system through a band inversion between spin‐polarized *a*_1*g*_ and $e_{g}^{\prime }$ V‐3d states. The band inversion is determined by an upward energy shift of the $e_{g}^{\prime }$ states in the valence bands and the crossing of the *a*_1*g*_ states from the conduction to the valence bands, so that both *a*_1*g*_ and $e_{g}^{\prime }$ states fall into the valence bands with an inverted energy position.

Interestingly, unlike the phase transition driven by internal correlation and external stimuli in other monolayers of transition metal trihalides, half‐metallic and insulating phases in VCl_3_ could be classified by Fermi level manipulation. For example, in OsCl_3_ exhibiting the SGS phase,^[^ ^37^ ^]^ correlation‐driven topological phase transition from the QAH to the Mott insulating phase is implemented by closing and reopening the bandgap in the manifold of Os t_2*g*_ bands crossing the Fermi energy. However, the distinction between a half‐metallic phase and an insulating phase in VCl_3_ is more related to the Fermi level position than the closing and reopening of the bandgap. That is, the low‐energy manifold of V‐d bands across the Fermi energy is characterized by a spin‐polarized Dirac point in both the half‐metallic and insulating phases. When the dispersing Dirac bands cross the Fermi level, the system is classified as a half‐metal.^[^ ^14^, ^28^, ^29^, ^30^ ^]^ However, when the Dirac bands lie below the Fermi level, with a valence band maximum (VBM) touching the Fermi level at the M‐point, the system is classified as an insulator.^[^ ^32^, ^36^ ^]^ More recently, using first‐principles methods, L. Camerano et al.^[^ ^34^, ^35^ ^]^ showed that the monolayer VCl_3_ realizes a ground state with multi‐component magneto‐orbital order. The multi‐component ground state with simultaneous magnetic and orbital ordering explicitly demonstrates the underlying symmetry‐determined origin of the half‐metallic and insulating phases in monolayer VCl_3_. The distribution of low‐energy d orbitals, the symmetry determination of the ground state, and the entropy‐driven effects on the electronic phases of VCl_3_ are further elaborated in Section 2.3 which explicitly demonstrates the orbital‐resolved band dispersion.

Based on our first‐principles calculations, consistent with the underlying symmetry subject to the Fermi‐level pinned at zero‐energy level (*E*_*F*_ = 0), the pristine VCl_3_ monolayer is a ferromagnetic DHM;^[^ ^28^, ^29^ ^]^ characterized by a Dirac dispersion in the spin‐up channel and a large indirect bandgap of >4.5 eV in the spin‐down channel, and an intrinsic long‐range ferromagnetic character with estimated Curie temperatures up to 425 K.^[^ ^29^ ^]^ Due to its DHM character, VCl_3_ is predicted to exhibit the QAH effect.^[^ ^28^, ^38^, ^39^ ^]^ However, despite all these interesting electronic and magnetic properties that are supposed to provide an ideal playground for the QAH effect, the VCl_3_ monolayer does not guarantee a robust QAH phase, which is evident from the spin‐polarized band dispersion, as shown in Figure 1c,e. The Dirac point lying above the Fermi level at valley momenta (K/K′) is accompanied by bulk states crossing the Fermi level at the Γ‐point. This behavior has also been confirmed through the non‐vanishing density of states at the Fermi level.^[^ ^29^ ^]^ As a result, as shown in the Figure 1e, SOI opens a nontrivial bandgap at valleys but the bulk states at the Γ‐point are further lowered and thus mix with the chiral edge states lying inside the nontrivial QAH gap. That is, even though spin‐orbit coupled VCl_3_ monolayer exhibits the QAH phase with a SOI‐induced bandgap of 29 meV in the vicinity of valleys,^[^ ^28^ ^]^ the QAH phase in VCl_3_ monolayer does not remain robust due to mixing of edge states with dissipative bulk states, a problem that also remains inevitable in magnetic doped topological insulators.^[^ ^2^, ^7^, ^40^ ^]^

We also performed first‐principles calculations for a 2 x 2 supercell of VCl_3_ monolayer that is comprised of four unit cells, as shown in Figure 1a. The electronic and magnetic properties of VCl_3_ 2 x 2 supercell remain same as that obtained for VCl_3_ primitive cell, as shown in Figure S1 (Supporting Information). That is, spin‐polarized band dispersion of VCl_3_ 2 x 2 supercell also exhibits a ferromagnetic Dirac half‐metallic character, though with several additional bands. In addition, consistent with calculations performed over VCl_3_ primitive cell, SOI opens a nontrivial QAH bandgap at valley momenta, which is the same in magnitude to that obtained for VCl_3_ primitive cell. However, similar to the VCl_3_ primitive cell, bulk states in the vicinity of Γ‐point lie inside the QAH gap. It reveals that the band dispersion of VCl_3_ 2 x 2 supercell is mere a folding of VCl_3_ 1 x 1 unit cell. This was further confirmed by unfolding the 2 x 2 band structure to reproduce the original VCl_3_ band structure by using a VASP band unfolding procedure. The consistency of folding and unfolding of band dispersion in momentum‐space is consistent with extrapolation in the real‐space, i.e., 2 x 2 supercell of VCl_3_ is simply an extrapolation of VCl_3_ unit cell along x‐ and y‐directions, as shown in Figure 1a.

### 2.2 Entropic V_0.5_(TiCrFeCo)_0.5_Cl_3_ Monolayer

**Figure 3:**
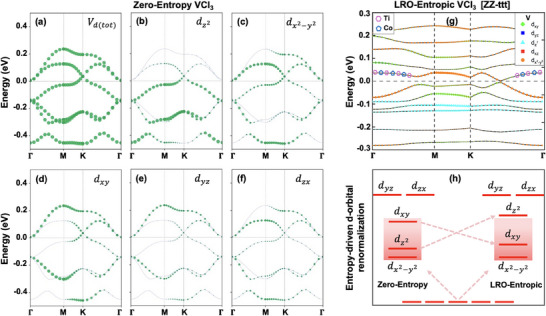
2D orbital‐resolved electronic dispersion and bandstructure renormalization. a–f) Orbital‐resolved band dispersion of pristine VCl_3_ monolayer (1 x 1 unit cell) showing total contributions of 3d‐orbitals of vanadium (a), contribution from *e*_*g*_ ($d_{z^{2}}$, $d_{x^{2}-y^{2}}$) orbitals of vanadium (b,c), and the contribution from *t*_2*g*_ (d_*xy*_, d_*yz*_, d_*zx*_) orbitals of vanadium (d,e,f). g) Orbital‐resolved band dispersion of LRO‐entropic VCl_3_ monolayer in ZZ‐ttt configuration (2 x 2 supercell) showing the contribution of 3d‐orbitals of vanadium (V) and M′ cations (Ti and Co). h) A schematic representation of entropy‐driven band‐inversion between d_*xy*_ and $d_{z^{2}}$ leading to the renormalization of vanadium 3d‐orbital. The size of the bands represents the orbital weight.

In the LRO‐entropic VCl_3_ with LRO along zigzag chains [ZZ‐ttt], as shown in Figure 1b and 2c, the M′ atoms are incorporated along zigzag chains such that the lattice structure is constituted by adjacent zigzag chains made of V‐atoms and M′‐atoms (Ti, Cr, Fe, Co) respectively. The geometric optimization of entropic V_0.5_(TiCrFeCo)_0.5_Cl_3_ 2 x 2 supercell shows that the optimized lattice constants remain the same as that of pristine VCl_3_ 2 x 2 supercell. However, the atomic positions/coordinates of both TM and Cl atoms are considerably changed. As shown in Figures 1 and **3**, the entropy‐driven bandstructure renormalization is featured by several interesting aspects such as band flattening, red and blue shifts at different momenta of the Brillouin zone, and a momentum shift of Dirac points. In the absence of SOI, Figures 1c and 3g, band flattening is a consequence of a blue shift in the vicinity of high‐symmetry Γ‐point and valleys K/K′ while a red shift around the M‐point and momenta along the K−Γ symmetry line. While the blue shift opens a trivial gap at the Dirac points K/K′, the red shift at momenta k and k′ along the symmetry lines K−Γ and K′ − Γ induces Dirac points at the Fermi level. As a result, Dirac points move away from valleys (K/K′) indicating an entropy‐driven renormalization of crystal field effects.

In addition, unlike the zero‐entropy VCl_3_ monolayer in which the Dirac point lies above the Fermi level at the K‐point while additional bulk states cross the Fermi level at the Γ‐point, the Dirac point in the LRO‐entropic VCl_3_ monolayer [ZZ‐ttt] lies exactly at the Fermi level (with a negligible gap of 6.6 meV) and the low‐energy states at the Γ‐point move away from the Fermi level. As a result, with an increase in entropy, VCl_3_ monolayer is transformed from a DHM to a DSGS. The remarkable aspect of doping is that, while the entropy of the material has increased, the fermi energy has decreased, sustaining the SGS Dirac point. This indicates that the electron density would have decreased. This would likely be a result of the electron localization around the doped ions. This is confirmed through the calculated Fermi energy and the bader charges.

**Figure 4:**
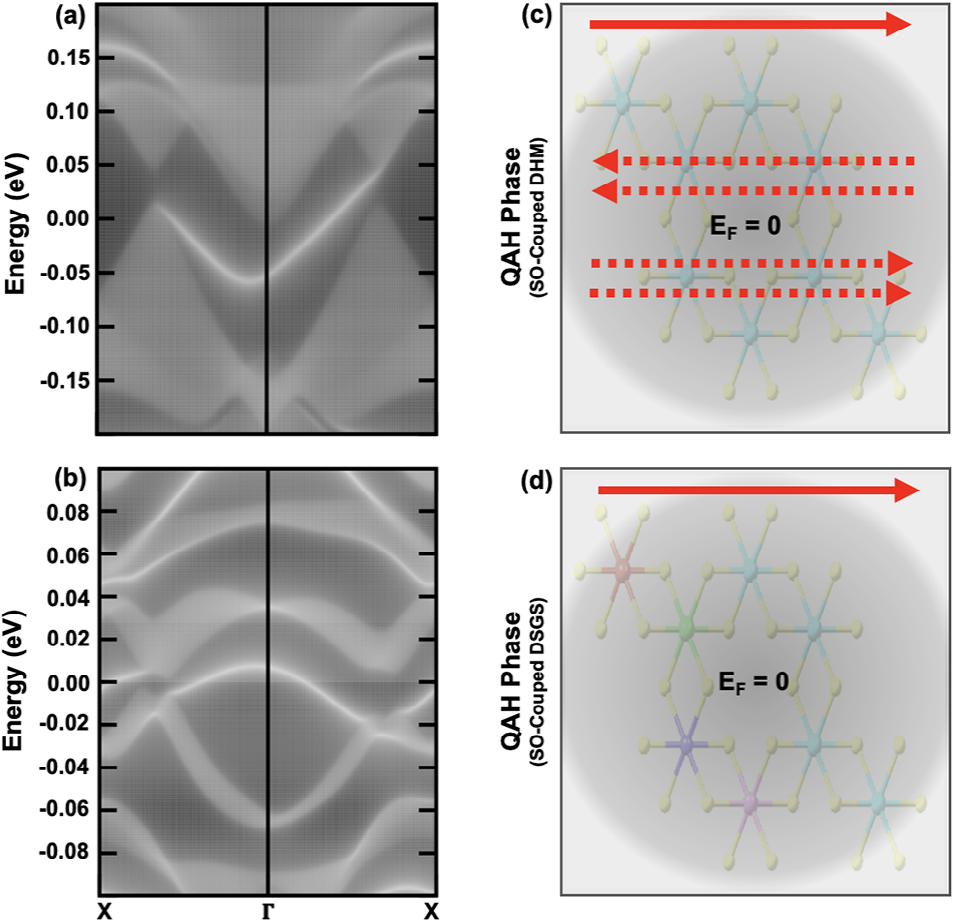
1D electronic dispersion and bulk‐boundary correspondence characterizing QAH effect. a, b) 1D band structure of zero‐entropy VCl_3_ monolayer (1x1 unit cell) (a) and LRO‐entropic VCl_3_ [ZZ‐ttt] monolayer (2x2 supercell) (b) showing the chiral edge state in momentum space along $\bar{X}-\Gamma -X$. c,d) A real‐space schematic representation of the chiral edge state (solid arrow) and bulk modes (dashed arrow) crossing the Fermi level *E*_*f*_ = 0 in zero‐entropy VCl_3_ monolayer (c) and LRO‐entropic VCl_3_ [ZZ‐ttt] monolayer (d). 1D dispersion clearly shows that the chiral edge states are mixed with the bulk modes in the zero‐entropy VCl_3_ monolayer (a,c), while the chiral edge states are completely disentangled from the bulk modes in the LRO‐entropic VCl_3_ [ZZ‐ttt] monolayer (b,d), a key feature of entropy‐engineering leading to a robust QAH effect.

SOI opens a nontrivial bandgap at the k/k′ points along the K/K′ − Γ symmetry line featuring the QAH effect, as shown in Figure 1e and Figure S2 (Supporting Information). The nontrivial topological character of SOI driven bandgap could be supported by various established mechanisms. First, the emergence of chiral edge states in 1D dispersion, **Figure** **4**b, validates the nontrivial bulk‐boundary correspondence characterizing bulk states by non‐vanishing Chern number C=±1. Second, the origin of QAH is due to the intrinsic topology of the honeycomb lattice structure terminating on the zigzag edges.^[^ ^41^ ^]^ In the absence of SOI, the intrinsic topology of pristine honeycomb leads to zero‐energy edge states connecting the two valleys K and K′, while the bulk spectrum remains gapless. A finite SOI, acting as a perturbation, opens a gap in the 2D bulk spectrum and disperses the zero‐energy modes into chiral edge states in the 1D edge‐state spectrum. As shown in Figure 4, the chiral edge states connecting the two valleys confirm the intrinsic origin of the QAH effect in honeycomb lattice structures terminating on the zigzag edges. Third, the underlying physics of the QAH effect in spin‐orbit coupled DSGSs is the same as that of the spinfull Haldane model,^[^ ^2^, ^7^ ^]^ where SOI acts as a Haldane‐type next‐nearest‐neighbor tunneling (λτσ_*z*_),^[^ ^6^ ^]^ a single copy of KM‐type SOI (λτσ_*z*_ *s*_*z*_) in honeycomb lattice structures. As a result, the SOI‐driven bandgap in the DSGSs leads to the spin QAH phase. Fourth, since the spin‐gapless dispersion of the LRO‐entropic VCl_3_ monolayer [ZZ‐ttt] is mainly constituted by vanadium d‐orbitals around the Dirac point(s), the origin of the QAH effect is also consistent with the previously reported QAH effect in the zero‐entropy VCl_3_ monolayer.^[^ ^28^, ^38^, ^39^ ^]^

Interestingly, unlike the half‐metallic VCl_3_ monolayer in which the spin‐polarized Dirac dispersion at the valley momenta is accompanied by bulk states at the Γ‐point and thus the QAH phase does not remain fully gapped, Figure 4a, the LRO‐entropic VCl_3_ monolayer [ZZ‐ttt] allows a fully gapped QAH phase and thus a purely topological edge state transport without mixing with dissipative bulk channels. The realization of DSGS phase and the robustness of corresponding QAH phase are associated with an entropy‐driven band flattening in combination with red and blue shifts at different momenta of the Brillouin zone. However, unlike a blue shift in the absence of SOI, an increase in entropy induces a red shift around valley momenta in the spin‐orbit coupled case. In addition, SOI has to overcome a gap of 6.6 meV in the pristine case before entering the QAH phase, more like a phase transition from magnetic semiconductor to the QAH phase. As a result, the size of the nontrivial bandgap in the entropic case is smaller than the bandgap in the zero‐entropy case. In a fully gapped QAH phase in LRO‐entropic VCl_3_, the bulk bandgap could be tuned via a suitable selection of TM atoms with the assurance of a blue shift at both Γ and K/K′ points.

To further investigate entropy‐driven bandstructure renormalization and the effects of symmetry breaking, LRO‐entropic cases SL‐t′t′t′, Mix‐t′t′t, and Mix‐t′tt are also analyzed. As shown in Figure 2d–f, along with the redistribution of TM atoms, nearest‐neighbor hopping matrix elements are also modified. As a consequence, the electronic dispersion also gets drastically modified from that of the LRO‐entropic ZZ‐ttt case. As shown in Figure S2 (Supporting Information), the LRO‐entropic chiral structure SL‐t′t′t′ displays a gapped dispersion also in the spin‐up sector and thus featuring a ferromagnetic insulating phase, Mix‐t′t′t displays a nodal‐line semi‐metallic character between nearly flat spin‐up and spin‐down low‐energy bands, while Mix‐t′tt is a ferromagnetic metal with a nearly flat spin‐up band across Fermi level. The Mix‐t′t′t configuration, with a nodal‐line semi‐metallic character, could also exhibit the QAH phase due to a Rashba‐type SOI.^[^ ^2^ ^]^ A finite Rashba SOI could potentially be allowed through a renormalization of crystal field effects due to a modified atomic arrangement of different TM‐atoms. Further details on the electronic and magnetic properties of these LRO‐entropic cases are discussed in the Supplementary Information.

### 2.3 Orbital‐Resolved Band Dispersion

In order to understand the microscopic origin of entropy‐driven bandstructure renormalization, especially band flattening leading to the transition from half‐metallic character to spin gapless semiconducting behavior and the accompanied effects on ferromagnetic ground state, orbital resolved band dispersion of LRO‐entropic V_0.5_(TiCrFeCo)_0.5_Cl_3_ monolayer [ZZ‐ttt] is investigated, as shown in Figure 3. Due to the entropy‐induced symmetry breaking, the bandstructure renormalization could originate from various effects at the microscopic level such as energy‐splitting and redistribution of V‐3d orbitals, contribution of spin‐up electrons from the substituted TM‐dopants, p‐d hybridization between orbitals of Cl‐atoms and TM‐atoms, and the contribution of various TM‐atoms to the total moment of magnetic ground state.

The pristine structure of VCl_3_ stabilizes in ferromagnetic ground state with a total magnetic moment of ≈4.0 µ_*B*_ per unit cell, where each V atom contributes ≈2.0 µ_*B*_ while the magnetic moment of the surrounding Cl atoms is negligible. The ferromagnetic nature remains persistent for the 2 × 2 supercell displaying a total magnetic moment of ≈16.0 µ_*B*_. These electronic and magnetic properties of the pristine monolayer VCl_3_ are consistent with previously reported first‐principle calculations.^[^ ^28^, ^29^, ^30^ ^]^ Like the half‐metallic VCl_3_ monolayer, the ground state of the LRO‐entropic monolayer [ZZ‐ttt] remains ferromagnetic; however, the total magnetic moment is reduced to 10.9729 µ_*B*_. The reduction in total magnetic moment is consistent with a phase transition from the DHM phase to a DSGS phase, in an itinerant ferromagnetic ground state.

As shown in Figure 3a–f and Figure S3 (Supporting Information), the intrinsic half‐metallicity in the VCl_3_ monolayer is dominated by the vanadium 3d orbitals, with a negligible contribution from the Cl‐p orbitals. The low‐energy Dirac bands are predominantly occupied by *e*_*g*_ orbitals ($d_{z^{2}}$ and $d_{x^{2}-y^{2}}$) and a *d*_*xy*_ orbital with a contribution from the *d*_*yz*_ and *d*_*zx*_ orbitals around the Γ‐point. The orbital contribution to low‐energy Dirac bands is in good agreement with previous first‐principles studies on the VCl_3_ monolayer.^[^ ^28^, ^30^ ^]^ In addition to octahedral crystal field splitting of 3d‐orbitals into a doublet *e*_*g*_ ($d_{z^{2}}$ and $d_{x^{2}-y^{2}}$) and a triplet *t*_2*g*_ (*d*_*xy*_, *d*_*yz*_ and *d*_*zx*_), transition‐metal trihalide monolayers with P31m symmetry could exhibit further splitting in the *t*_2*g*_ triplet such that low energy states are contributed by a singlet $e_{out}\to a_{1g}∼d_{z^{2}}$ and a doublet $d_{in}\to e_{g}^{\prime }∼\left(d_{xy},d_{x^{2}-y^{2}}\right)$.^[^ ^34^, ^35^, ^42^ ^]^

Like zero‐entropy VCl_3_ monolayer, spin‐up bands of LRO‐entropic monolayer [ZZ‐ttt] are also predominantly occupied by a doublet $d_{in}\to e_{g}^{\prime }$ and a singlet *e*_*out*_ → *a*_1*g*_, as shown in Figure 3g. However, there are several interesting modifications induced by enhanced configurational entropy. First, the low‐energy Dirac bands are constituted by doublet $d_{in}\to e_{g}^{\prime }$ while the contribution of *e*_*out*_ → *a*_1*g*_ to the Dirac bands is completely depleted. It shows that only in‐plane 3d orbitals ($d_{in}\to e_{g}^{\prime }$) contribute to the spin‐up Dirac bands around the Fermi level, while the contribution from 3d‐orbitals that extend along the out‐of‐plane direction (*d*_*yz*_, *d*_*zx*_, $d_{z^{2}}$) is diminished with an increase in the entropy, leading to a red shift around the M‐point and along the K‐Γ line. The entropy‐driven redistribution of 3d‐orbitals is depicted in Figure 3h. Second, the contribution from V atoms to a low‐energy conduction band is significantly reduced at the Γ‐point. On the other hand, as displayed in Figure 3g and Figure S4b,c (Supporting Information), contribution of Ti and Co to the low‐energy conduction band is prominent at the Γ‐point. However, as shown in Figure S4d,e (Supporting Information), the contribution from Fe and Cr atoms is vanishingly small to the low‐energy valence bands. As a result, the bulk states move away from the Fermi level; a blue shift at the Γ‐point, leading to the DHM‐DSGS phase transition. Such an entropy‐driven depletion of V‐3d orbitals with weight along the z‐axis and a finite contribution from doped M′ atoms (Ti and Co) causes a band flattening due to red and blue shifts at various momenta of the Brillouin zone.

The entropy‐driven redistribution of 3d‐orbitals and the accompanied red and blue shifts leading to the band flattening, is consistent with a symmetry‐identified ground state in VCl_3_ monolayer.^[^ ^34^, ^35^ ^]^ In the octahedral environment *O*_*h*_, the Jahn–Teller distortion^[^ ^43^ ^]^ lowers the symmetry from *O*_*h*_ to the trigonal point group *D*_3*d*_, i.e. splitting the low‐energy manifold in a singlet *e*_*out*_ → *a*_1*g*_ and a doublet $d_{in}\to e_{g}^{\prime }$. A spontaneous symmetry breaking could completely eliminate orbital degeneracy by further splitting the $d_{in}\to e_{g}^{\prime }$ manifold into $e_{g,1}^{\prime }$ and $e_{g,2}^{\prime }$. First‐principles DFT+U (U = 3.2 eV) calculations showed that the ground state of the pristine VCl_3_ monolayer with *V* ^3 +^ (3*d* ^2^) configuration could be stabilized in the metallic phase $a_{1g}e_{g,1}^{\prime }$ and the insulating phase $e_{g,1}^{\prime }e_{g,2}^{\prime }$, both giving rise to magneto‐orbital ordering.^[^ ^34^ ^]^ In the present case, as shown in Figure 3h, ground state of zero‐entropy VCl_3_ monolayer exhibits the metallic phase $a_{1g}e_{g,1}^{\prime }$ while the ground state of LRO‐entropic V_0.5_(TiCrFeCo)_0.5_Cl_3_ monolayer displays a small gap spin‐gapless semiconducting phase $e_{g,1}^{\prime }e_{g,2}^{\prime }$. The entropy‐driven phase transition from half‐metallic to DSGS phase is in good agreement with the symmetry‐identified half‐metallic and insulating ground states.

In addition, unlike the zero‐entropy VCl_3_ monolayer, a contribution of Cl‐p orbitals is enhanced in the LRO‐entropic [ZZ‐ttt] monolayer, as shown Figure S4f (Supporting Information), leading to a hybridization between spin‐up d‐orbitals of TM atoms and spin‐down p‐orbitals of Cl atoms. Such p‐d hybridization has also been predicted in Mn‐ and Fe‐doped VCl_3_ monolayers.^[^ ^30^ ^]^ The p‐d hybridization can further be validated by inspecting a reduction in magnetic moments of V‐3d orbitals. In the zero‐entropy VCl_3_ monolayer, our first principle calculations show that each V^3 +^ [3*d* ^2^] atom contributes a magnetic moment of *m*_*B*_ = +1.918 µ_*B*_ while each Cl^−^ atom contributes *m*_*B*_ ≈ −0.03 µ_*B*_, as shown in Table S1 (Supporting Information). In the LRO‐entropic monolayer [ZZ‐ttt], on the other hand, while two of V atoms contribute *m*_*B*_ ≈ +1.90 µ_*B*_, the magnetic moment of the other two V atoms reduces to *m*_*B*_ ≈ +1.80 µ_*B*_ and that of Cl atoms varies from *m*_*B*_ ≈ −0.001 µ_*B*_ to *m*_*B*_ ≈ −0.03 µ_*B*_.

The diverse nature of magnetic coupling between the TM‐3d orbitals and the surrounding Cl‐p orbitals also affects the magnetic moments of TM‐atoms, leading to a ferromagnetic ground state with a total magnetic moment of *m*_*B*_ ≈ +10.9729 µ_*B*_, much smaller than the magnetic moment of zero‐entropy case (≈16 µ_*B*_). Interestingly, unlike an antiferromagnetic coupling Cl↓‐TM↑‐Cl↓ between the local magnetic moments of Cl atoms and TM atoms (V, Ti, Co, and Cr), a negative value of the local magnetic moment of the Fe cation indicates a ferromagnetic coupling Cl↓‐Fe↓‐Cl↓ of the Fe atom with neighboring Cl atoms. This shows that entropy engineering could also be a promising design concept to control magnetic textures in real space. It has been predicted through first‐principle calculations that the electronic and magnetic properties of VCl_3_ monolayer can be tuned through single‐atom substitutional doping with 3d TM atoms (Sc, Ti, Cr, Mn, Fe).^[^ ^30^ ^]^ However, as shown in Table S2 (Supporting Information), it is crucial to note that TM atoms doped in a LRO‐entropic environment exhibit characteristics that are distinct from the doping of individual TM atoms in zero‐entropy VCl_3_. Further details about the magnetic moments of individual TM atoms and local spin textures for various LRO‐entropic configurations are summarized in the Supplementary Information.

The renormalization of electronic and magnetic properties of LRO‐entropic V_0.5_(TiCrFeCo)_0.5_Cl_3_ monolayer [ZZ‐ttt] shows that an interplay between electrons localization and de‐localization emerges as a trademark of entropy engineering. While band flattening suggests that the decrease in the bandwidth is a direct consequence of entropy manipulation that reduces the inter‐orbital overlap, a considerable contribution from Cl atoms and the reduced magnetic moments of TM atoms constituting low‐energy bands indicate a hybridization between the d‐orbitals of TM atoms and the p‐orbitals of the surrounding Cl atoms. It shows that the LRO‐entropic monolayer [ZZ‐ttt] is a special case in the sense that nearest‐neighbor hopping on the zigzag chains made of V‐atoms predominantly constitutes low‐energy Dirac bands, featuring an intrinsic topological characteristic of honeycomb structures, while the entropy level raised by doped M′ atoms reduces the bandwidth of Dirac bands. The interplay between electrons localization and de‐localization is also evident from the electronic and magnetic properties of other LRO‐entropic configurations. For example, in the Mix‐t′t′t configuration with a total magnetic moment of *m*_*B*_ ≈ +3.286 µ_*B*_, an interplay between localization and de‐localization leads to the band dispersion that exhibits a nodal‐line semi‐metallic character between nearly flat spin‐up and spin‐down bands, as shown in Figure S2b (Supporting Information). On the other hand, in the SL‐t′t′t′ configuration with a total magnetic moment of *m*_*B*_ ≈ +12.143 µ_*B*_, localization dominates over de‐localization leading to a band dispersion exhibiting ferromagnetic insulating behavior, as shown in Figure S2a (Supporting Information). This shows that entropy engineering is an effective mechanism for controlling the bandstructure through an interplay between electrons localization and de‐localization.

## 3 Conclusion and Outlook

Bandstructure renormalization and energy splitting of low‐lying orbitals through entropy engineering are attributed to structural symmetry breaking caused by TM dopants in transition‐metal trihalides.^[^ ^30^, ^36^ ^]^ In addition, apart from the level of entropy and the nature of symmetry breaking, the electronic and magnetic properties of entropic materials rely on what elements are contributing to the low‐energy bands and how their low‐lying orbitals are influenced by the nearest‐neighbor environments. In order to understand the impact of the nearest‐neighbor environment on the electronic and magnetic properties, we systematically changed the sublattice site position of doped TM atoms with parent V atoms while keeping the level of entropy fixed. Our first‐principle calculations show that a small change in the nearest‐neighbor environment could lead to a significant change in electronic and magnetic properties.

The bandstructure renormalization in all four configurations of the long‐range ordered V_0.5_(TiCrFeCo)_0.5_Cl_3_ monolayer is evidently an entropy‐driven effect, directly related to an increase in the number of dopants (entropy) and the nearest‐neighbor hopping (local environment). However, the ZZ‐ttt configuration is special in the sense that it exhibits quantum phases that feature an intrinsic topological character of honeycomb lattice structures. A much relevant and needed question to be addressed could be which of these four long‐range ordered configurations is the most favorable to achieve thermodynamic stabilization. In biatomic systems, the SL‐t′t′t′ configuration could be more favorable due to structural chirality, as demonstrated recently in the Mn/V‐doped CrI_3_ crystals, i.e., CrMnI_6_^[^ ^44^ ^]^ and CrVI_6_,^[^ ^45^ ^]^ which have been experimentally synthesized based on DFT predictions. However, the ZZ‐ttt configuration could be more favorable in multi‐component systems because of the intrinsic momentum‐space topology and its affects on phonon‐dispersion in honeycomb structures. A further study would be required to comprehensively address this question based on the intrinsic topological character of the honeycomb lattice structures, the nearest‐neighbor environment and the electronic configuration of doped TM atoms quantifying the hopping amplitudes, and a thorough investigation of the thermal history and phonon dispersions for accessing stability.

A robust QAH effect is a unique feature of SGSs,^[^ ^2^, ^7^ ^]^ i.e., SOI opens a nontrivial energy gap by lifting the only twofold degeneracy of the Dirac point(s) in the bulk band dispersion that corresponds to the fully spin‐polarized chiral modes in the edge state spectrum. In 2D magnets, the electronic transition from ferromagnetic DHM to a ferromagnetic DSGS and thus a robust QAH effect could open a new route to revolutionize low‐energy and high‐performance electronic and spintronic technologies, such as giant magnetoresistance devices^[^ ^2^, ^4^, ^46^ ^]^ overcoming the Schmidt obstacle^[^ ^47^, ^48^ ^]^ and topological switching devices^[^ ^2^, ^49^, ^50^, ^51^ ^]^ overcoming the Boltzmann tyranny.^[^ ^2^, ^41^, ^52^, ^53^, ^54^, ^55^ ^]^ Although the entropy‐driven QAH phase is robust, i.e., chiral edge states are completely disentangled from the bulk modes, the QAH gap in entropic VCl_3_ is smaller than the QAH gap in zero‐entropy VCl_3_. This reduction in the nontrivial bandgap is consistent with the role of SOI and the Berry curvature which capitalize on their maximal strength at valley points. It motivates further research to find suitable TM atoms that could reduce the crystal‐field effect and enhance the nontrivial bandgap via blue shift at Dirac points, leading to a room‐temperature realization of topological quantum device applications.

VCl_3_ is a rich system that hosts emergent quasiparticles such as polarons,^[^ ^32^ ^]^ skyrmions,^[^ ^56^ ^]^ and magneto‐orbitons via magneto‐orbital coupling.^[^ ^34^ ^]^ It has also been demonstrated, via first‐principle calculations combined with nonequilibrium Green's function, that a magnetic tunnel junction VCl_3_/CoBr_3_/VCl_3_ made of ferromagnetic half‐metallic VCl_3_ exhibits a perfect spin filtering effect and a high tunnel magnetoresistance ratio (≈4.5 × 10^12^%).^[^ ^14^ ^]^ Moreover, a coexistence of ferroelectricity and magnetism has recently been observed in a VCl_3_ monolayer selectively grown on an NbSe_2_ substrate.^[^ ^33^ ^]^ In addition, like several other 2D materials with honeycomb lattice structures such as 2D‐Xenes^[^ ^57^, ^58^ ^]^ and van der Waals (vdW) heterostructures constructed by transition metal dichalcogenides and transition metal halides,^[^ ^59^ ^]^ VCl_3_ also hosts valley‐polarized QAH states due to the coexistence of QAH and QVH effects.^[^ ^38^, ^39^ ^]^ In zigzag nanoribbons of honeycomb lattice structures, because of an intrinsic topology and a nontrivial Berry curvature intertwined with valley DOF, spin‐valley coupling and the valley‐polarized states promise a wide range of applications in spin electronics and valley electronics. Considering the proposed proof‐of‐concept robustness of the QAH effect via entropy engineering, it would be interesting to explore how entropy engineering could further enhance the splitting of the valley polarization state to realize the robust valley‐polarized QAH effect in a wider range of material systems.

The proposed concept for materials design and discovery of DSGSs through entropy engineering, employed here for VCl_3_ monolayer, could also be extended for other 2D DHMs and 2D Dirac materials. For instance, like VCl_3_ monolayer, intrinsic magnetism,^[^ ^36^ ^]^ spin gapless electronic structure, and the corresponding SOI‐induced QAH effect could be renormalized in various other monolayers of transition metal trihalides such as OsCl_3_,^[^ ^37^ ^]^ RuX_3_ (X:Br,Cl,I),^[^ ^60^ ^]^ MnX_3_ (X:F,Cl,Br,I),^[^ ^61^ ^]^ NiCl_3_,^[^ ^62^ ^]^ PtCl_3_,^[^ ^63^ ^]^ PdCl_3_,^[^ ^64^ ^]^ MBr_3_ (M:Pd,Pt),^[^ ^65^ ^]^ MBr_3_ (M:V,Fe,Ni,Pd),^[^ ^66^ ^]^ FeX_3_ (X:Cl,Br,I),^[^ ^67^ ^]^ and VX_3_ (X:Cl,I).^[^ ^14^, ^28^, ^29^, ^30^ ^]^ Similarly, the proposed design concept could also be employed for enhancing the robustness of QAH phase in magnetic‐doped topological insulators, i.e., dissipative bulk channels, and thus backscattering could potentially be removed by increasing the configurational entropy.

Recently, single crystalline VCl_3_ has been grown^[^ ^32^ ^]^ using the self‐selecting vapor growth (SSVG) technique and structural characterization of the as‐grown crystals is presented by energy‐dispersive X‐ray (EDX) analysis and X‐ray photoemission spectroscopy (XPS). In addition, a structural phase transition at T = 103 K and an antiferromagnetic transition at *T*_*N*_ = 21.8 K are observed through temperature‐dependent heat capacity measurements using the Evercool II Quantum Design Physical Property Measurement System (PPMS). The nature of magnetism and the role of symmetry breaking is also demonstrated through experimental observation and first‐principles calculations in a recently synthesized VCl_3_‐NbSe_2_ heterojunction.^[^ ^33^ ^]^ In an epitaxially grown VCl_3_ monolayer on an NbSe_2_ substrate, vdW interfacial Cl‐Se interactions break both in‐plane *C*_3_ rotational and out‐of‐pane inversion symmetries, inducing in‐plane ferroelectricity accompanied by antiferromagnetic order with canted magnetic moments. Scanning tunneling microscopy (STM) and scanning tunneling spectroscopy (STS) provide consistent evidence that the VCl_3_ monolayer hosts in‐plane ferroelectricity while a bistriped antiferromagnetic order (*T*_*N*_ = 16 K) with an easy‐plane *xz* is confirmed by vibrating sample magnetometry (VSM). In addition, based on DFT predictions, biatomic transition‐metal trihalide CrVI_6_^[^ ^45^ ^]^ has recently been experimentally synthesized. The experimental progress and confirmation of emergent functionalities in vanadium‐based trihalides motivate the synthesis of an entropic VCl_3_ monolayer where entropy engineering could play an elegant role in the tailoring of crystalline symmetries and the corresponding emergent quantum phases.

Compared to single‐atom substitutional doping in the VCl_3_ monolayer,^[^ ^30^ ^]^ thermodynamic phase stability is more favorable in an entropic VCl_3_ monolayer. The configurational entropy is correlated with the phase transition and phase stabilization. That is, a phase transition from a thermodynamically unstable phase to a thermodynamically stable phase is associated with entropy‐driven phase stabilization.^[^ ^68^ ^]^ The general concept of entropy‐dominated phase stabilization is based on the reduction in the Gibbs free energy Δ*G* (Δ*G* = Δ*H* − *T*Δ*S*), predominantly by increasing the entropy Δ*S* of the system. This can be done by introducing multiple elements with random distribution at the same lattice sites.

The topological and magnetic properties can change with temperature. Although, the origin of the nontrivial topology in the entropic V_0.5_(TiCrFeCo)_0.5_Cl_3_ monolayer is intertwined with the intrinsic topological character of honeycomb lattice structures,^[^ ^41^ ^]^ the temperature dependence of the band topology in the entropic V_0.5_(TiCrFeCo)_0.5_Cl_3_ monolayer can change from that for the pristine VCl_3_ structure, mainly due to entropy‐driven effects on phonon dispersion and an intimate coupling between phonons and various emergent electronic and magnetic quasiparticles. On the same grounds, the Curie temperature and its temperature dependence in the V_0.5_(TiCrFeCo)_0.5_Cl_3_ monolayer could be different from those in the VCl_3_ monolayer. Furthermore, as shown in the supplementary information, first‐principles analysis of the temperature dependence of the Curie temperature becomes more demanding in these entropic configurations, in which magnetic moments of TM atoms are sensitively dependent on the nearest‐neighbor environment. A comprehensive analysis on the temperature dependence of the band topology and Curie temperature in the V_0.5_(TiCrFeCo)_0.5_Cl_3_ monolayer is beyond the scope of the present study, which presents a proof‐of‐concept for tailoring a robust QAH effect via entropy engineering. Considering the fundamental importance of the performance stability of materials in high‐temperature environments, a detailed analysis on the temperature dependence of topological and magnetic properties in entropic V_0.5_(TiCrFeCo)_0.5_Cl_3_ monolayer would pave the way for practical applications.

## Conflict of Interest

The authors declare no conflict of interest.

## Author Contributions

M.N. and X.W. conceived the project idea, designed the research, and co‐wrote the manuscript. S.A. and F.F. performed the first‐principles calculations and participated in the analysis and discussion of the results.

## Supporting information

Supporting Information

## Data Availability

The data that support the findings of this study are available from the corresponding author upon reasonable request.
