# Supplementary material for: Tailoring Robust Quantum Anomalous Hall Effect via Entropy‐Engineering
Source: Adv Mater. 2025 Jun 3;37(36):2503319. doi: 10.1002/adma.202503319 (PMC12422079; doi:10.1002/adma.202503319)
Supplement: Supplementary file 1 — Supporting Information [file ADMA-37-2503319-s001.pdf]

ADVANCED MATERIALS

Supporting Information

for *Adv. Mater.*, DOI 10.1002/adma.202503319

Tailoring Robust Quantum Anomalous Hall Effect via Entropy-Engineering

Syeda Amina Shabbir, Frank Fei Yun, Muhammad Nadeem and Xiaolin Wang**

Tailoring Robust Quantum Anomalous Hall Effect via Entropy-Engineering [SUPPLEMENTARY INFORMATION]

Syeda Amina Shabbir Frank Fei Yun Muhammad Nadeem Xiaolin Wang**

1 Electronic dispersion and the role of entropy

Band dispersion of zero-entropy VCl_3 monolayer, 1 x 1 unit cell and 2 x 2 supercell, and entropic VCl_3 monolayer with long-range order (LRO) along the zigzag chains [ZZ-ttt] is shown in figure **S1**. Unlike half-metallic character in zero-entropy monolayers, figure **S1**(a-d), ZZ-ttt entropic configuration exhibits spin gapless semiconducting (SGS) behavior, where SOI opens a nontrivial energy gap leading to a fully gapped quantum anomalous Hall (QAH) phase, figure **S1**(e,f). Figure **S2** shows band dispersion for other three LRO-entropic configurations, namely SL-t′t′t′], Mix-t′t′t, and Mix-t′tt.
In the LRO-entropic chiral structure [SL-t′t′t′], TM-atoms are substituted on the sublattice sites such that the triangular sublattice A is formed by V atoms and the triangular sublattice B is formed by M′ (Ti, Cr, Fe, Co) atoms. In this configuration, an increase in entropy modifies nearest neighbor hopping between V atoms and the dopant M′ atoms such that a gap is induced in the low-energy bands. As a consequence, the LRO-entropic chiral structure displays a gapped dispersion featuring a ferromagnetic insulating phase, as shown in figure **S2**(a). Consistent with previous analysis for the ZZ-ttt configuration, entropy engineering drives band flattening along with a blue shift at K/K′ points while a red shift across M-point. However, the bulk states across the Γ-point remain mostly unaffected, indicating a redistribution of various orbitals' contributions to the low-energy bands and the associated crystal fields. Furthermore, unlike the ZZ-ttt configuration that displays an intertwining between localization and de-localization of d-orbitals, localization dominates over de-localization in the SL-t′t′t′ configuration. On the other hand, in the Mix-t′t′t LRO entropic configuration, an interplay between localization and de-localization leads to the band dispersion that exhibits a nodal-line semi-metallic character between nearly flat spin-up and spin-down bands, as shown in figure **S2**(b). Interestingly, a small change in this configuration, induced by exchanging a sublattice site of one of the V atoms with a Cr atom, drastically changes the band dispersion, as shown in figure **S2**(c). This shows that entropy engineering is an effective mechanism for controlling the bandstructure.
Detailed orbital resolved band dispersion of zero-entropy VCl_3 monolayer (1 x 1 unit cell) and the LRO-entropic VCl_3 monolayer ZZ-ttt (2 x 2 unit cell) are shown in figure **S3** and **S4**, respectively. In the zero-entropy VCl_3 monolayer, the low-energy conduction band of the Dirac dispersion is predominantly occupied by the $d_{x^2-y^2}$ orbital, along with a contribution from the d_{xy} and d_{xy} orbitals along the M-K-Γ and K-M-Γ line, respectively. On the other hand, low-energy valence band of the Dirac dispersion is predominantly occupied by the d_{z^2} orbital, along with a small contribution from in-plane 3d orbitals ($d_{x^2-y^2}$ and d_{xy}) around the Dirac point. However, in the LRO-entropic ZZ-ttt configuration, the low-energy Dirac bands are completely occupied by the in-plane 3d orbitals ($d_{x^2-y^2}$ and d_{xy}) while the 3d orbitals with out-of-plane component (d_{z^2}, d_{yz}, d_{zx}) do not contribute to the states around the Fermi level in the spin-up channel. Orbital contributions from doped M′ atoms are also depicted in figure **S4**.

2 Magnetic moments and local spin textures

As shown in tables 1 and 2, local magnetic moments and local spin textures strongly depend on the level of entropy and the nearest-neighbor environment. In addition, the entropy-induced transformation of the ferromagnetic ground state, as well as the modification of electronic structure, can also be understood based on the differences between the number of valence electrons contributed by the M′ atoms and those from the V atoms. For instance, similar to the ferromagnetic ground state of the VCl_3 monolayer with a total magnetic moment of 4 μ_B (16 μ_B for a 2 x 2 supercell), the ground states of the LRO-entropic monolayers ZZ-ttt and SL-t′t′t′ remain ferromagnetic, but the total magnetic moment reduces to 10.972

μ_B and 12.143 μ_B, respectively. In addition, local magnetic moments of individual TM cations and local spin textures strongly depend on the level of entropy and the type of LRO.

In the LRO-entropic monolayer ZZ-ttt, similar to the high-spin state of vanadium V^{3+} [$3d^2$], the magnetic moments of Ti ($m_B \approx +0.413\ \mu_B$) and Cr ($m_B \approx +2.738\ \mu_B$) are found to be consistent with their respective values in the +3 oxidation state, i.e., Ti^{3+} [$3d^1$] and Cr^{3+} [$3d^3$]. However, the magnetic moments of Co ($m_B \approx +0.582\ \mu_B$) suggest that it is found in the low-spin state, Co^{2+} [$3d^7$]. On the other hand, the Fe atom contributes $m_B \approx -0.743\ \mu_B$ to the total magnetic of the ZZ-ttt configuration, which is oppositely aligned to the magnetic moments of other TM cations. It suggests that Fe is found to be in the low-spin state Fe^{3+} [$3d^5$] and the Cl↓-Fe↓-Cl↓ bond favors ferromagnetic interactions. This behavior of Fe in the LRO-entropic monolayer ZZ-ttt is completely different from that of Fe cations in Fe-doped low-entropy VCl_3 [1], where Fe^{3+} [$3d^5$] is found to exist in a high-spin state ($m_B \approx +4.106\ \mu_B$), enhancing the total magnetic moment of the VCl_3 2 x 2 monolayer from 16 μ_B to 19 μ_B, and the ferromagnetic nature of Cl↑-Fe↑-Cl↑ bond is indebted to the positive value of the local magnetic moment of the Cl atoms. The local spin texture of Fe and the deviation from a high-spin state in the low-entropy case to a low-spin state in the high-entropy case could be a consequence of the level of entropy and symmetry-breaking effects.

In the SL-t′t′t′ configuration, low/high spin states of the TM cations remain the same as in the ZZ-ttt configuration. However, unlike the ZZ-ttt configuration, the magnetic moments of all the TM cations remain positive, and thus an antiferromagnetic interaction is favored by all the Cl↓-TM↑-Cl↓ bonds, as indicated by the negative local magnetic moments of the Cl atoms. That is, the local spin textures are similar to that in the zero-entropy case. However, the magnetic moments of V atoms are further reduced from the zero-entropy case.

Local magnetic moments and local spin textures become more interesting in the LRO-entropic Mix-t′t′t and Mix-t′tt configurations. First, the magnetic moments of two of the V atoms are significantly reduced to +0.393 μ_B and +0.253 μ_B in the Mix-t′t′t configuration. Second, like the ZZZ-ttt and SL-t′t′t′ configurations, Fe and Cr favor a low-spin state and a high-spin state, respectively. However, the local magnetic moments of both Fe and Cr are negative. Third, unlike the ZZZ-ttt and SL-t′t′t′ configurations, the magnetic moments of Co ($m_B \approx +2.455\ \mu_B$) suggest that it is found in a high-spin state, Co^{2+} [$3d^7$]. In the LRO-entropic Mix-t′tt, on the other hand, the magnetic moment of only one of the V atoms is reduced to +0.421 μ_B while the other three shows $m_B \approx +1.9\ \mu_B$. Interestingly, the reduced magnetic moment of that V atom appears to be negative, highly contrasting from other LRO-entropic configurations. In addition, while the magnetic moment of the low-spin Fe state remains negative, the magnetic moment of the high-spin Cr state becomes positive. Furthermore, unlike Mix-t′t′t but like ZZZ-ttt and SL-t′t′t′ configurations, the magnetic moments of Co ($m_B \approx +0.597\ \mu_B$) suggest that it is found in the low-spin state, Co^{2+} [$3d^7$]. The magnetic ground state of Mix-t′t′t and Mix-t′tt configurations stabilizes with a reduced total magnetic moment of $m_B = +3.286\ \mu_B$ and $m_B = +8.747\ \mu_B$, respectively. In both of these mix entropic configurations, positive magnetic moments for some of the Cl atoms while negative magnetic moments for the other Cl atoms suggest an intermingling of ferromagnetic and antiferromagnetic Cl↑↓-TM↑↓-Cl↑↓ bonds.

A momentous change in the local magnetic moments and spin textures in the LRO-entropic Mix-t′t′t and Mix-t′tt configurations show that the electronic and magnetic properties are significantly altered by the relocation of a single Cr atom, indicating the fragile impact of entropy engineering on electronic and magnetic properties.

References

[1] C. Ouettar, H. Yahi, K. Zanat, H. Chibani, *Physica Scripta* **2023**, *98*, 2 025814.

Sample	M_{V_1}	M_{V_2}	M_{V_3}	M_{V_4}	M_{V_5}	M_{V_6}	M_{V_7}	M_{V_8}	M_{Cl}	M_{total}
VCl_3(1x1)	1.935 ↑	1.935 ↑	-	-	-	-	-	-	∼ 0.03 ↓	4↑
VCl_3(2x2)	1.916 ↑	1.916 ↑	1.916 ↑	1.916 ↑	1.916 ↑	1.916 ↑	1.916 ↑	1.916 ↑	∼ 0.03 ↓	16↑

Table 1: Magnetic moments and spin orientations of V-atoms in zero-entropy VCl_3 monolayer. All values are given in μ_B.

Sample	M_{V_1}	M_{V_2}	M_{V_3}	M_{V_4}	M_{Ti}	M_{Fe}	M_{Cr}	M_{Co}	M_{Cl}	M_{total}
ZZ-ttt	1.918 ↑	1.876 ↑	1.822 ↑	1.783 ↑	0.413 ↑	0.743 ↓	2.738 ↑	0.582 ↑	(0.001-0.034)↓	10.972 ↑
SL-t′t′t′	1.863 ↑	1.849↑	1.779↑	1.697↑	0.485 ↑	0.533 ↑	2.748 ↑	0.552 ↑	(0.001-0.036)↓	12.143 ↑
Mix-t′t′t	1.827↑	1.762↑	0.393↑	0.253↑	0.166 ↑	0.963 ↓	2.713 ↓	2.455↑	(0.001-0.035)↓↑	03.286 ↑
Mix-t′tt	1.948↑	1.946↑	1.902↑	0.421↓	0.402 ↑	0.849 ↓	2.777 ↑	0.597 ↑	(0.001-0.033)↓↑	08.747 ↑

Table 2: Magnetic moments and spin orientations of TM cations in LRO-entropic $TiV_4CrFeCoCl_{24}$ monolayers. Shaded cells represent an entropy-driven transition in spin up/down polarization and/or low/high spin states of TM atoms and the appearance of Cl atoms in both spin-up and spin-down states. All values are given in μ_B.

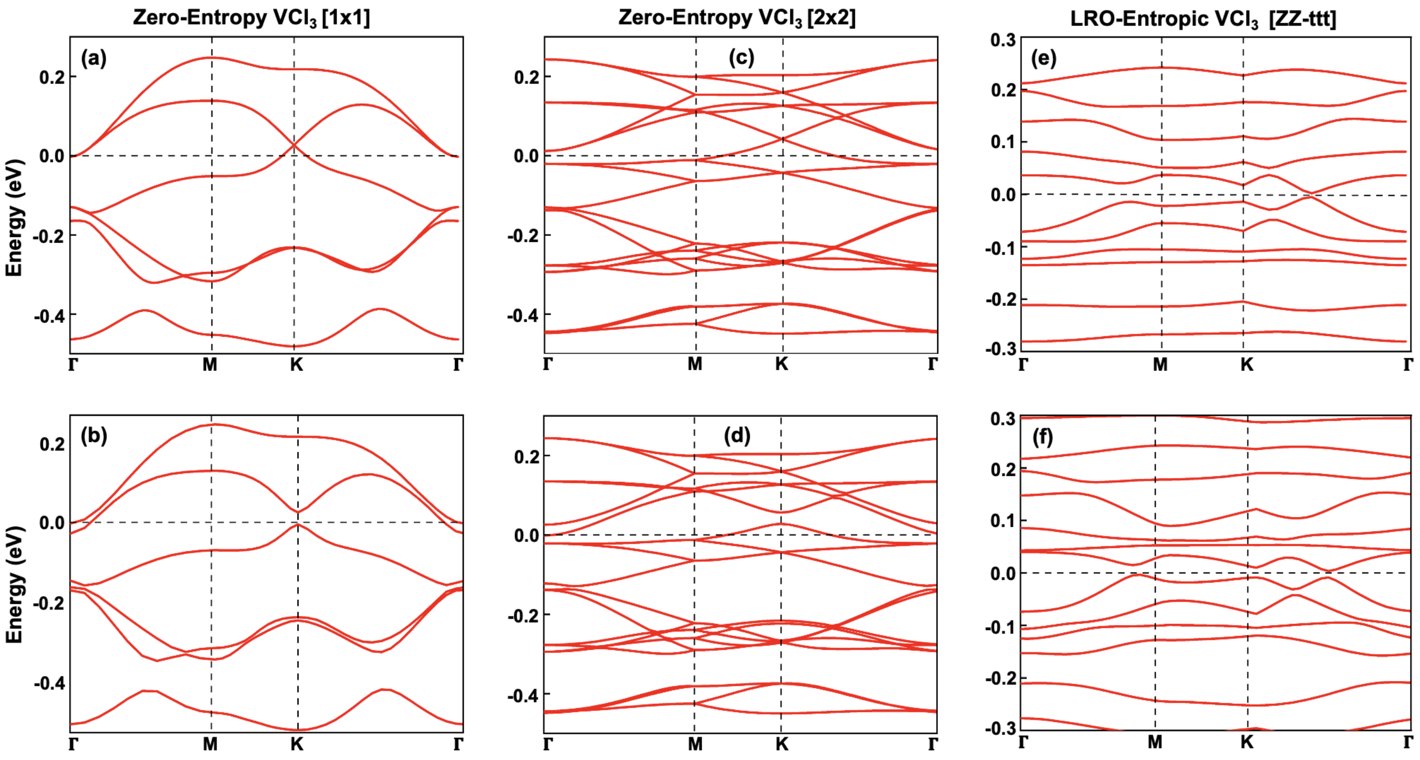

Figure 1: **[S1:] Electronic dispersion of vanadium trichloride monolayer. (a,b)** Band dispersion of zero-entropy VCl_3 monolayer (1 x 1 unit cell) without SOI (a) and with SOI (b). **(c,d)** Band dispersion of zero-entropy VCl_3 monolayer (2 x 2 supercell) without SOI (c) and with SOI (d). **(e,f)** Band dispersion of entropic $TiV_4CrFeCoCl_{24}$ monolayer (2 x 2 supercell), with a long-range order along zigzag chains, without SOI (e) and with SOI (f).

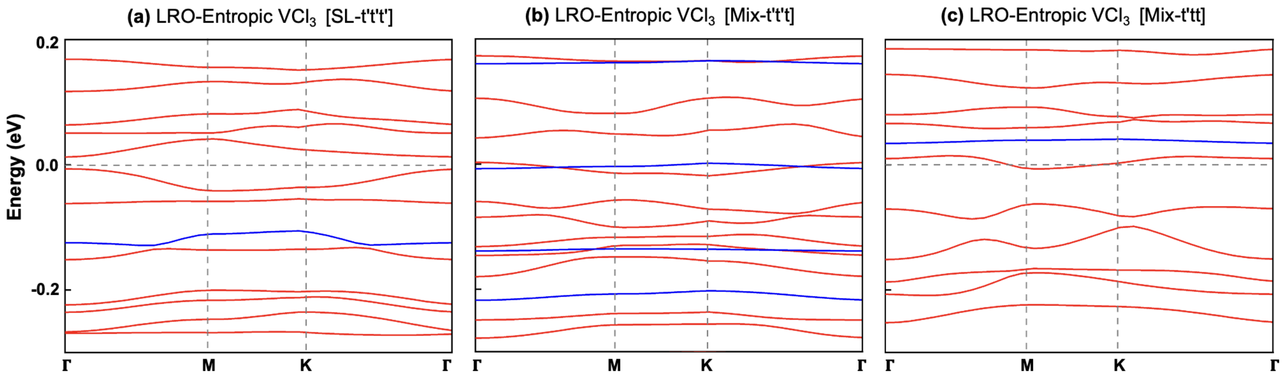

Figure 2: **[S2:] Nearest neighbor effect on the electronic dispersion of LRO entropic VCl_3 monolayer.** Band dispersion of LRO entropic VCl_3 monolayer [SL-t′t′t′] (a) [Mix-t′t′t] (b), and [Mix-t′tt. Here red and blue bands represent spin-up and spin-down sectors, respectively.]

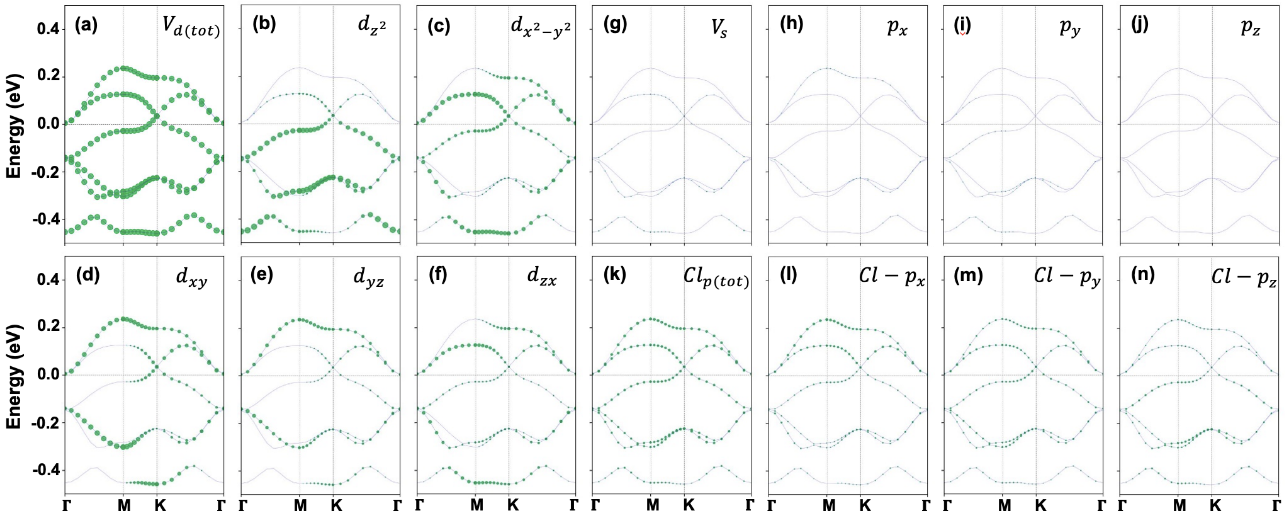

Figure 3: **[S3:] Orbital resolved band dispersion of VCl_3 monolayer. (a-f)** Orbital contribution from d-orbitals of vanadium (a), e_g orbitals (d_{z^2}, $d_{x^2-y^2}$) (b,c) and t_{2g} orbitals (d_{xy}, d_{yz}, d_{zx}) (d,e,f). **(g-j)** Orbital contribution from s-orbitals (g) and p-orbitals (h,i,j) of vanadium. **(k-n)** Orbital contribution from p-orbitals of Cl. The size of the bands represents the orbital weight.

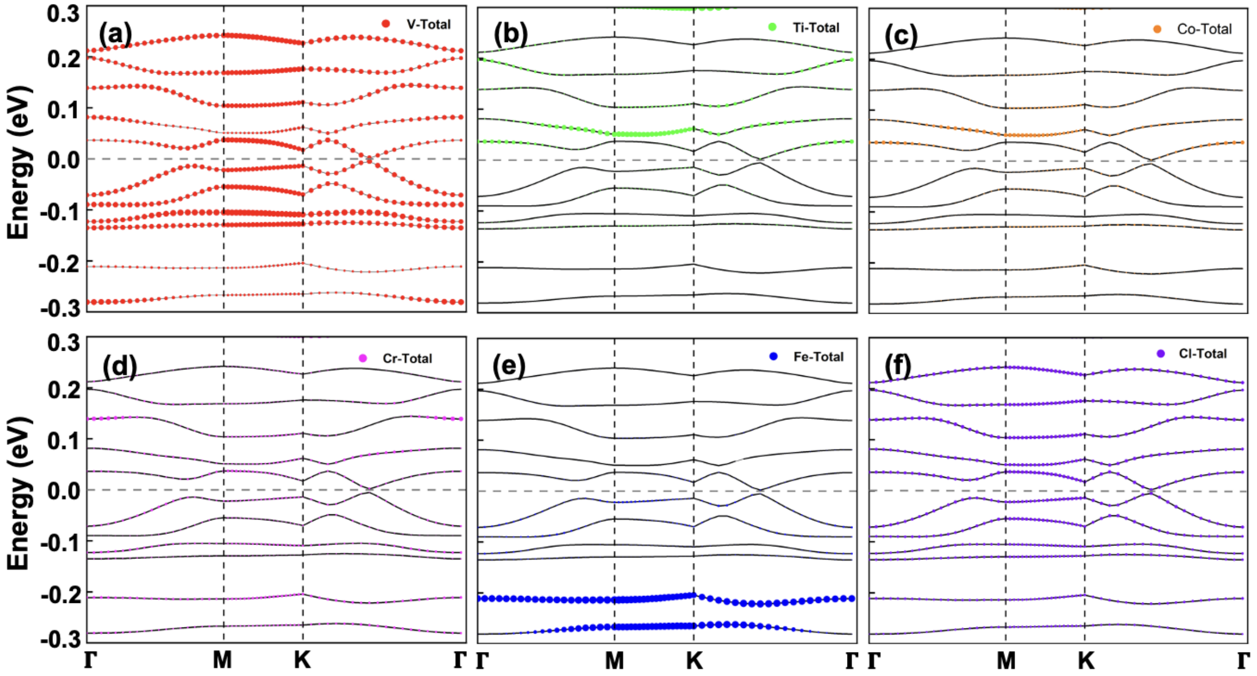

Figure 4: [**S4:**] **Orbital resolved band dispersion of entropic VCl_3 monolayer. (a-f)** Orbital-resolved band structures in $TiV_4CrFeCoCl_24$ monolayer with the corresponding contribution from V (a), Ti (b), Co (c), Cr (d), Fe (e), and Cl (f) atoms, respectively. The size of the bands represents the orbital weight.
